# Linking Susceptibility to Infectious Diseases to Immune System Abnormalities among HIV-Exposed Uninfected Infants

**DOI:** 10.3389/fimmu.2016.00310

**Published:** 2016-08-19

**Authors:** Candice Ruck, Brian A. Reikie, Arnaud Marchant, Tobias R. Kollmann, Fatima Kakkar

**Affiliations:** ^1^Department of Pediatrics, BC Women’s and Children’s Hospital, University of British Columbia, Vancouver, BC, Canada; ^2^Department of Surgery, University of Manitoba, Winnipeg, MB, Canada; ^3^Institute for Medical Immunology, Université Libre de Bruxelles, Charleroi, Belgium; ^4^Department of Pediatrics, CHU Sainte-Justine, Université de Montréal, Montréal, QC, Canada

**Keywords:** HIV exposure, infectious diseases, morbidity, mortality, immunology

## Abstract

HIV-exposed uninfected (HEU) infants experience increased overall mortality from infectious causes when compared to HIV-unexposed uninfected (HU) infants. This is the case in both the resource-rich and resource-limited settings. Here, we explore the concept that specific types of infectious diseases that are more common among HEU infants could provide clues as to the potential underlying immunological abnormalities. The most commonly reported infections in HEU vs. HU infants are caused by encapsulated bacteria, suggesting the existence of a less effective humoral (antibody, complement) immune response. Decreased transplacental transfer of protective maternal antibodies has consistently been observed among HEU newborns, suggesting that this may indeed be one of the key drivers of their susceptibility to infections with encapsulated bacteria. Reassuringly, HEU humoral response to vaccination appears to be well conserved. While there appears to be an increase in overall incidence of acute viral infections, no specific pattern of acute viral infections has emerged; and although there is evidence of increased chronic viral infection from perinatal transmission of hepatitis C and cytomegalovirus, no data exist to suggest an increase in adverse outcomes. Thus, no firm conclusions about antiviral effector mechanisms can be drawn. However, the most unusual of reported infections among the HEU have been opportunistic infections, suggesting the possibility of underlying defects in CD4 helper T cells and overall immune regulatory function. This may relate to the observation that the immunological profile of HEUs indicates a more activated T cell profile as well as a more inflammatory innate immune response. However, both of these observations appear transient, marked in early infancy, but no longer evident later in life. The causes of these early-life changes in immune profiles are likely multifactorial and may be related to *in utero* exposure to HIV, but also to increased environmental exposure to pathogens from sicker household contacts, *in utero* and postnatal antiretroviral drug exposure, and, in certain circumstances, differences in mode of feeding. The relative importance of each of these factors will be important to delineate in an attempt to identify those HEU at highest risk of adverse outcomes for targeted interventions.

## Background

Due to successful interventions to prevent mother-to-child transmission (PMTCT) of HIV, the risk of HIV transmission in resource-rich settings has been reduced to <1% (1). The successful scale-up of PMTCT programs in low- and middle-income countries is leading to transmission rates approaching those seen in resource-rich settings and means that, worldwide, HIV-exposed uninfected (HEU) infants represent a growing proportion of the pediatric population. In certain HIV-endemic nations, HEU infants represent nearly 30% of the newborn population (2). While this decrease in vertical transmission of HIV represents a tremendous achievement, it is not without consequence, for, while these infants are not infected with HIV, they are affected by the virus and by the treatments given during pregnancy to prevent transmission (3).

The first cohorts that followed HEU children longitudinally have reported increased morbidity and mortality when compared to HIV-unexposed uninfected (HU) children, and specifically, increased severity of infectious diseases (reviewed by Slogrove et al. in this Research Topic) (4, 5). In certain HIV-endemic countries with high prevalence of maternal HIV infection, it is estimated that over half of all childhood mortality in the first 24 months of life may be due to HIV exposure (6). While the cause of this increased morbidity and mortality from infectious diseases among HEU infants is likely to be multifactorial, encompassing environmental, maternal, and health systems factors, the increased burden of infections seen among them raises the question of whether immune alterations may contribute to their increased susceptibility to disease. To date, a number of studies have shown evidence of various immunological abnormalities among HEU children (7), with alterations in both humoral and cell-mediated immunity (CMI) reported. It is hypothesized that these immunological changes may result from a combination of factors whereby the *in utero* environment of HIV-infected mothers uniquely shapes their infant’s immune system, leading to an increased susceptibility to infectious diseases.

In view of the number of recent reports of infectious diseases among HEU children, the main objective of this review is to probe the existing data regarding the infectious pathogens observed in HEU infants, and their association to specific alterations in immune defense mechanisms, in an effort to better understand the increased susceptibility to infectious disease observed in this vulnerable population.

## Part 1: Clinical Findings among HEU Infants

### Rates of Mortality among HEU Infants

Beginning as early as 2003, the first cohorts to follow HEU children reported increased morbidity and mortality when compared to HU children (8). While the overall mortality rate in studies of HEU infants varies (ranging from 4.6 to 18.7% in the African setting, see Table 1) (7–16), the majority of studies have demonstrated increased mortality among HEU vs. HU infants across all settings, with mortality rates ranging from twice to fourfold above HU controls. Moreover, it appears that the cause of mortality, when investigated, is predominantly infectious. Specifically, studies in Botswana and Durban, South Africa, demonstrated higher rates of treatment failure in HEU infants diagnosed with pneumonia when compared to HU infants, with higher associated mortality (17, 18). HEU infants also suffered higher mortality from invasive pneumococcal disease (IPD) when compared to HU infants (33.7 vs. 22.4%) in a South African surveillance study (19) and increased mortality from lower respiratory tract infection (OR: 2.1, CI: 1.1–3.8) compared to HU infants (20). The emerging pattern is one of increased mortality from infectious diseases, and primarily from respiratory illness, among HEU infants.

**Table 1:** **Mortality among HEU vs. HU infants**.

	Setting	HEU (*n*)	HU (*n*)
Marinda et al. (13)	Zimbabwe	9.2% (3135)	2.9% (9210)
Shapiro et al. (9)	Botswana	6.7% (534)	1.7% (137)
Brahmbhatt et al. (10)	Uganda	16.5% (269)	12.8% (3183)
Van Der Loeff et al. (8)	Gambia	14% (64)	8% (448)
Taha et al. (21)	Malawi	4.6% (439)	3.6% (108)
Sutcliffe et al. (12)	Zambia	5% (108)	1% (211)
Chilongozi et al. (14)	Multisite Africa	7.2% (302)	4.8% (1429)
Kelly et al. (17)	Botswana	12.5% (64)	2.6% (153)
Landes et al. (15)	Malawi	18.7% (173)	4.3% (214)
Ajibola et al. (16)	Botswana	5.1% (453)	1.8% (457)

### Rates of Hospitalization/Illness

In addition to increased overall mortality, recent studies have reported increased rates of all-cause hospitalization among HEU children when compared to HU children. Among 825 HEU children in the European Collaborative Study, 25% had been hospitalized in the first 2 years of life, with a reported rate of 0.5 per 5 child-years (22). A study of 736 HEU infants in India found that 35% of HEU infants had been hospitalized within the first year of life, with an overall rate in infancy of 906 per 1000 person-years (PY) (23). Again in that study, the majority (56%) of hospitalizations were due to infectious diseases (primary three causes included acute gastroenteritis 18.6%, sepsis/meningitis 11.5%, and pneumonia 6.6%). This pattern of high incidence of hospitalization has also been observed in resource-rich settings. In Belgium, the incidence of severe infections was estimated at 16.8% HEU infant years (24). In France, the risk of serious infections during the first year of life was estimated at 9.3% in HEU children (25). In a Canadian cohort, a higher rate of hospitalization was seen among infants born to mothers with detectable viral load at delivery (31 per 100 PY) compared to mothers with undetectable viral load (10 per 100 PY) (*p* = 0.02) (26). In addition to hospitalization, increased health-care utilization among HEU infants has been described. In one of the largest longitudinal studies of HEU children in Zimbabwe, sick clinic visits were 1.2 times more likely among HEU compared to HU children (27). Again, where documented, the overwhelming cause of health-care visits/hospitalizations was due to infectious diseases. In a small prospective South African cohort, HEU infants were 3.6 times more likely to develop a severe infection requiring hospitalization in their first year of life compared to HU controls (28). In a laboratory-based surveillance study in South Africa, HEU infants were found to have twice the rate of hospitalization from IPD compared to HU infants (19). A prospective South African cohort study of children undergoing surgery observed significantly higher rates of postsurgical complications among HEU compared to HU controls (23.7 vs. 5.7%); these were most often surgical site complications (impaired wound healing, infection, breakdown) or sepsis (29). In a large series from the Caribbean, HEU infants were found to have three times the incidence of neonatal infections compared to HU infants (26 per 1000 vs. 8.1 per 1000) (30). Taken together, these studies indicate an increased rate of health-care visits, hospitalization, and severe infections in the first 2 years of life among HEU infants across all settings.

### Specific Infectious Diagnosis among HEU Children

Amidst the reports of increased morbidity, mortality, and hospitalization among HEU children, a number of studies have documented the causative pathogens, whose presence may suggest specific areas of immune dysfunction (Table 2).

**Table 2:** **Infectious diagnosis and immune correlates among HEU infants**.

	Country	Findings	Infant immune correlates	Maternal immune correlates	Reference
**Fungal infections**					
*Pneumocystis jirovecii*	United States 1997	Two cases PJP	Transient decrease in absolute CD4 cell count (cases 1 and 2). Normal immunoglobulin and lymphocyte proliferation (case 2)	Maternal CD4 counts 27 and 47 cells/mm^3^	Heresi et al. (31)
*Pneumocystis jirovecii*	South Africa 2001–2002	Three cases of PJP	Not assessed	Not assessed	McNally et al. (18)
*Pneumocystis jirovecii*	South Africa 2010	Three cases of PJP	1 case persistently low CD4 count, 1 case transiently low, 1 case normal. Immunoglobulin levels normal (2), not assessed (1)	None WHO stage 3 or 4 disease. Median CD4 count 538 cells/mm^3^	Slogrove et al. (32)
*Pneumocystis jirovecii*	South Africa 2006–2008	Five cases of PJP	Not assessed	Not assessed	Morrow et al. (33)
*Candida*	Zimbabwe 1997–2000	Increased incidence of oral candidiasis	Not assessed	Increased incidence in infants of mothers with CD4 <200 cells/mm^3^ (IR 3.91) vs. <800 cells/mm^3^ (IR 1.91)	Evans et al. (34)
**Bacterial infections**					
*Streptococcus pneumoniae*	South Africa 2009–2013	Increased rate of invasive pneumococcal disease HEU vs. HU (33–88 per 100,000 vs. 18–28 per 100,000)	Not assessed	Not assessed	Von Mollendorf et al. (19)
*Streptococcus pneumoniae*	Belgium 1985–2006	Fourfold increase in rate in HEU vs. HU	Not yet available, studies underway	Twofold increase in frequency of severe infections with maternal CD4 count <200 (not statistically significant)	Adler et al. (24)
Group B *Streptococcus*	Belgium 2001–2008	13-fold increase in rate of invasive disease in HEU vs. HU	Increased total leukopenia at start of sepsis compared to HU infants	In one of six infants, maternal CD4 count <350 cells/mm^3^	Epalza et al. (35)
Group B *Streptococcus*	South Africa 2004–2007	2.25-fold increase in incidence of invasive disease in HEU vs. HU	Not assessed	Increased risk of early onset sepsis among infants of mothers CD4 <200 vs. >350 cells/mm^3^	Cutland et al. (36)
*Haemophilus influenzae, Streptococcus pneumoniae*	France 2002–2010	Encapsulated organisms were the cause of 56.7% of bacterial infections in infancy	No association between infant CD4 count at birth and risk of infection	Association between lower maternal CD4 count and serious bacterial, but not viral infections	Taron-Brocard et al. (25)
*Mycobacterium tuberculosis*	South Africa, Botswana 2004–2008	Incidence of pulmonary TB fourfold higher HEU than historical controls	Not assessed	Not assessed	Madhi et al. (37)
**Viral infections**					
*Respiratory (RSV, Rhinovirus, Enterovirus, Adenovirus)*	South Africa 2010–2013	Increased incidence causing LRTI among HEU vs. HU	Not assessed	Not assessed	Cohen et al. (20)
*Hepatitis B*	Malawi 2004–2009	11.9 and 12.8% HBV infection in infants of women HbsAg or HBV DNA positive	Not assessed	Not assessed	Chasela et al. (38)
*Cytomegalovirus*	United States 1998–2002	Increased rate of congenital CMV infection (3.6%) vs. general population (1%)	Not assessed	Increased risk of congenital or early postnatal CMV with maternal CD4 <200 vs. >200 cells/mm^3^	Frederick et al. (39)
*Epstein–Barr virus*	Kenya 1999–2003	Early acquisition of EBV infection	Not assessed	Maternal prenatal CD4 <20% and viral load >4.5-log RNA, associated with increased rate of EBV acquisition	Slyker et al. (40)

#### Fungal Infections

The most striking of infectious findings among HEU infants have been case series reporting *Pneumocystis jirovecii* pneumonia (PJP) in infancy. The earliest of these were two case reports in Texas of HEU infants diagnosed with PJP at <2 months of age (31). Subsequently, PJP was confirmed among three HEU infants in a cohort of infants with severe pneumonia in Durban, South Africa (18). A study of infants hospitalized with hypoxic pneumonia in Cape Town reported PJP in 5 of the 34 who were HEU (33), while a separate study also in South Africa found PJP in 3 of 8 hospitalized HEU infants with severe pneumonia (32). A large prospective cohort study in Zimbabwe (*n* = 3185) studying health outcomes in HEU infants found an increased incidence of oral candidiasis in HEU vs. HU infants (34). Fungal infections, and specifically PJP, are typically considered opportunistic infections seen in immune compromised individuals. Their presence is strongly suggestive of immunodeficiency and warrants thorough investigation of T lymphocyte function and number in particular (41, 42).

#### Bacterial Infections

Bacterial infections have also featured prominently in reports of increased morbidity and hospitalization of HEU infants. Among clinical cohorts with microbiologically confirmed diagnoses, the clearest difference in infectious pathogens between HEU and HU infants are infections caused by encapsulated bacteria. A cross-sectional study of infants with IPD in South Africa found a higher rate among HEU infants (33–88 per 100,000) compared to HU infants (18–28 per 100,000) (19). A Belgian study found that rates of IPD were fourfold higher among HEU infants compared to the general infant population (24). The same cohort also reported rates of group B *Streptococcus* (GBS) infection that were 13-fold higher in HEU infants compared to the general infant population (35). A hospital-based surveillance study in Soweto, South Africa, reported a 2.25-fold greater incidence of GBS in HEU compared to HU infants (36). In a large French cohort documenting severe infections among HEU infants during their first year of life, encapsulated bacteria (*Haemophilus influenzae* and *Streptococcus pneumoniae*) were the causative organism for 56.7% of confirmed bacterial infections (25). Susceptibility to encapsulated organisms may be due to defects in humoral immunity, complement deficiency, or asplenia. Antibody-mediated, or -humoral, immunity is particularly important in the defense against infection caused by polysaccharide-encapsulated bacteria. Infants are transiently protected from these infections through transplacental transfer of maternal antibody, until their own humoral responses develop. An increase in infections by such organisms in infancy would be suggestive of decreased maternal antibody protection, while an increased susceptibility to such infections after the age of two would be more suggestive of defects in humoral immunity (43). In addition to humoral immunity, another key defense against bacterial pathogens is the complement system, part of the innate immune response. Individuals with specific complement deficiency would have increased susceptibility to infection with these bacterial pathogens (44, 45). These infections would also be more common in individuals with congenital or acquired asplenia, such as those with underlying hemoglobinopathies (46).

Due to the high burden of tuberculosis (TB) coinfection among HIV-infected patients, a higher incidence of TB among HIV-infected pregnant women and subsequently their exposed infants would be expected when compared to HU controls. To date, however, there have been no comparative studies on the true incidence or severity of TB disease in HEU vs. HU infants. In a prospective randomized control trial of isoniazid prophylaxis among HIV-infected and HEU children in a South African cohort, the incidence of pulmonary TB was fourfold higher among HEU when compared to a historical control group of HUs in South Africa (37). Difficulties in obtaining microbiological confirmation of TB disease may potentially have resulted in an underreporting of TB-related deaths, likely classified as pneumonia. If indeed an increased incidence of severe TB disease is confirmed among HEU infants, this may be related to increased exposure to TB, or it may further suggest a defect in T cell function or number, and/or defects in interferon gamma (INF-γ) receptor pathways or tumor necrosis factor (TNF) signaling pathways (47, 48), which are necessary for control of mycobacterial infections.

#### Viral Infections

The frequency and severity of viral infections among the HEU infants has been more difficult to characterize, likely due to technical challenges and the costs associated with viral pathogen testing in resource-limited settings. While upper respiratory tract infections (URTIs) and LRTIs have been more frequently reported (30), only one study to date has characterized specific respiratory viral pathogens seen in HEU vs. HU children. In a South African surveillance study, there was an increased risk of all-cause LRTI (OR: 1.4, CI: 1.3–1.5) among HEU vs. HU, with increased incidence of respiratory syncytial virus (RSV), rhinovirus, enterovirus, adenovirus, and *Human metapneumovirus*-associated LRTI, but no difference in incidence of influenza (IRR: 1.2, CI: 0.8–1.8) (20). Among the common acute life-threatening systemic viral infections of childhood, there are surprisingly no reports of increased frequency of varicella or measles infections in the HEU children. CMI is the key mechanism of defense against viral pathogens. If indeed higher rates of acute viral illness among HEU were confirmed, this could be due to disorders of CMI, which would include both quantitative or qualitative deficiencies in T cells, or natural killer cells (NK cells), or antigen-presenting cells (APCs) (49, 50). At the same time, decreased transfer of maternal antibodies and deficiencies in innate immune function, through limitations in cytokine production or cytokine responsiveness, would also contribute to increased susceptibility to acute viral infections.

Among chronic viral diseases, the reported rates of perinatal Hepatitis C transmission is consistently higher among HEU than in the general population of HU infants (2.5-fold), likely due to increased maternal viremia and viral shedding (51). However, there is no evidence of increased perinatal hepatitis B virus (HBV) transmission (38), potentially due to increasing use of antiretroviral regimens with specific activity against HBV during pregnancy (tenofovir and emtricitabine). Finally, among latent infections, HIV-infected women have a much higher rate of transmission of cytomegalovirus (CMV), with a reported prevalence of congenital CMV of 1.5–7% among HEU infants, compared to only 1% among HU infants (39, 52, 53). The role of Epstein–Barr virus (EBV) infections in the morbidity and mortality of HEU infants is less clear. While primary EBV infection has been shown to occur early in HEU infants (mean time to primary infection 11 months) (40), there have been no studies comparing EBV acquisition and pathogenesis between HEU and HU infants. Effector CD4 T cells are important for the control of these chronic viral pathogens that infect cells for at least part of their life cycle and could be a factor in increased susceptibility to and symptoms associated with congenital herpes virus infections, such as CMV (54).

## Part II: Immune Alterations among HEU Children

### Adaptive Immune Responses

#### T Lymphocytes

The initial reports of PJP pneumonia – typically an opportunistic pathogen in individuals with defects in CMI – among HEU infants first prompted investigation into the T cell response of HEU infants. In five of the above cases of PJP where CD4 T cell number was assessed, three reported transient decreases in absolute CD4 T cell number with full recovery, one with persistently low CD4 count, and one case with normal CD4 count (31, 32). In one case, lymphocyte proliferation was assessed and was found normal (31).

Overall, phenotypic differences in T cell populations have been detected in multiple studies of HEU infants. A Brazilian study reported a neonatal immune profile skewed away from a naive phenotype, with decreased proportions of naive CD4 and CD8 T cells and increased proportions of central memory CD4 and CD8 T cells in HEU compared to HU children (55). HEU infants have also been shown to have higher T cell expression of activation marker CD38 (56). Similarly, Clerici et al. reported reduced proportions of naive CD4 and CD8 T cells and increased proportions of memory CD4 and CD8 T cells, combined with increased percentages of activated CD8 T cells in HEU compared to HU newborns (57). Lower absolute CD4 T cell counts have been reported among HEU infants exposed to higher levels of maternal viremia *in utero* (58), lower maternal CD4 T cell count (59), and those exposed to any antiretrovirals (60), suggesting that there may be individual differences in absolute CD4 T cell number within the HEU population. Taken together, these reports suggest *in utero* priming of T lymphocytes in HEU newborns. Whether this priming is related to exposure to specific antigens crossing the placenta or to non-antigen-specific activation remains to be determined, as are any direct clinical implications.

Functional differences in HEU lymphocyte populations have also been detected, although they have proven more inconsistent and therefore difficult to interpret. Cord blood mononuclear cells of HEU infants displayed reduced production of IL-4 and IL-7 and increased production of IL-10 and INF-γ compared to HU infants (61). However, these findings contrast those of an earlier study that reported significantly higher concentrations of serum IL-7 in HEU newborns, as well as older children, compared to their age-matched HU controls (57). There is a lack of data to explain this discrepancy, although in the former study, all HIV-positive mothers had low or undetectable viral load, while maternal viral load information was not available for the later study. Increased IL-10 production in response to phytohemagglutin (PHA) stimulation of cord blood leukocytes in HEU infants compared to HU was previously described in a South African cohort that took place in the pre-ARV era (62). This is in contrast to reports of reduced levels of IL-10 in the cord blood mononuclear cells of HEU infants whose mothers did not control viral load (63).

Functional responses to antigenic stimuli are also difficult to interpret, given the limited available data thus far. A study from South Africa reported that stimulation with *Staphylococcus Enterotoxin B* (SEB) and *Bacille Calmette–Guérin* (BCG) induced increases in CD4 and CD8 T cell proliferation; however, proliferation was comparable between HEU and HU in response to stimulation with *Bordetella pertussis* antigen (64). An investigation into markers of activation and cytokine expression at 3 and 12 months of age in response to tetanus toxoid in a Kenyan cohort did not detect a difference in TNF-α, IFN-γ, or IL-2 at 3 months of age (65).

In short, while reports of PJP pneumonia are suggestive of immune deficiency affecting T lymphocytes, the possible mechanisms are not yet well understood. While the induction of memory T cells has been consistently observed, it remains unclear whether *in utero* HIV exposure influences the magnitude or quality of T cell responses to pathogens or vaccines.

#### B Lymphocytes and Antibodies

The increased incidence of GBS and pneumococcal sepsis in infancy among HEU infants across multiple settings suggests increased susceptibility to bacterial pathogens in the first year of life. The most unequivocal difference in immune status reported among HEU and HU infants is the decreased transfer of maternal antibodies to HIV-exposed newborns. Actively transported maternal IgG are the source of virtually all the IgG subclasses detected in the fetus and neonates, and these maternally derived levels fall rapidly after birth, reaching a nadir of approximately 4000 mg/dL in term infants at 3–4 months of age (66). A number of studies have reported reduced levels of maternally derived antibodies to vaccine- or pathogen-specific antigens, including tetanus (67–70), measles (71, 72), *Haemophilus influenzae* B (Hib), pertussis and pneumococcus (69), and GBS in HEU as compared to HU newborns (Abu Raya et al. in review) (73). These reduced levels can be related to lower levels of specific antibodies in HIV-infected women, reduced placental transfer, or a combination of these. A Brazilian study found reduced levels of antibodies to tetanus, measles, and pneumococcus in HEU neonates, despite maternal antibody levels that were comparable between HIV-positive and HIV-negative mothers (74). Bashir et al. reported that HIV-positive women were 16.27 times more likely than HIV-negative women to be seronegative for anti-tetanus antibody, while their infants were 33.75 times more likely to be seronegative (67). They further concluded that HIV-positive mothers were 4.91 times more likely to have poorly efficient transplacental transfer of antibodies. A study in South Africa reported lower levels of antibodies to Hib and pneumococcus in HIV-infected women, but no significant difference for tetanus or pertussis (69). Levels of anti-GBS antibodies were found to be reduced in HIV-infected women compared to uninfected women in two separate South African cohorts (73, 75). Unfortunately, the studies documenting lower transplacental transfer of maternal antibody to HEU infants have not directly examined the clinical outcomes of these infants; thus, any association between lower antibody levels and increased risk of sepsis from encapsulated organisms remains a hypothesis. However, studies have shown an association between maternal immune suppression (lower CD4 T cell count) and increased incidence of bacterial infections (24, 25, 36), suggesting that maternal immune suppression, leading to decreased transplacental transfer of protective antibodies, represents a likely mechanism.

While studies are underway looking at humoral responses to vaccines in HEU infants, thus far, these appear to be intact. A few studies indicated that antibody responses to pertussis and pneumococcus vaccines were actually higher in HEU as compared to HU infants, whereas the responses to tetanus and Hib vaccines were similar in the two groups (68, 69). The higher vaccine response may be related to a lower inhibition by the reduced levels of maternal antibodies (76).

### Innate Immune Responses

Concurrent with decreased protection from maternal antibodies, differences in early-life innate immune development between HEU and HU infants may predispose them to increased infectious disease morbidity, most commonly from viral infections (76). Innate immunity orchestrates the initial, non-specific response to pathogens, while shaping future adaptive responses to prevent or clear infections (3) While there is very limited data on innate immune development among HEU infants, studies have shown that the first year of life is a period of rapid change in innate immune response, with differences in the developing immune profile of HEU vs. HU infants.

While well-known phenomena of neonatal innate immune development, such as deficiency in α and β defensins or complement pathways, may contribute to increased susceptibility to fungal infections in infancy, these may be magnified in HEU infants through additional mechanisms. Comparisons of early-life innate immunity in HEU and HU infants have demonstrated altered secretion of immune-mediating cytokines, with decreased IL-12 production in HEU vs. HU infants (77). Upregulation of cell surface receptors, such as increased MHCII expression, has been identified on unstimulated HEU APCs, relative to HU APCs (78). Functional comparison of NK cell activity at 1 month of age also demonstrated an increase of an intermediate NK phenotype for activation and perforin expression in HEU vs. HU, which was no longer significant by 1 year of age (79). Studies have also demonstrated a hyperinflammatory innate immune profile among HEUs and a proinflammatory innate immune response to stimulation with pathogen-associated molecular patterns (PAMPs) early in life. In a prospective study of innate immune development in the first year of life among HEUs, HEU APCs responded more strongly than HU in both the proportion of responder cells, and quantity of cytokine produced per cell, with a strong pattern of hyperresponsive polyfunctional monocytes and classical dendritic cells. The majority of differences were observed between 2 weeks and 6 months of age and were largely in response to bacterial PAMPs (80).

Different ethnic groups (with varied genetic backgrounds by extrapolation) can exhibit varied innate immune responses (81, 82). More specifically, TLR polymorphisms are associated with heterogeneity of innate responses (83). Therefore, in comparing HEU and HU immune development, it is essential to consider any differences in the ethnic distribution of populations where HIV is more vs. less prevalent. Future studies are needed to examine relative contributions of potential etiological factors to changes in innate immune ontogeny, and how those changes in immune development contribute to early-life susceptibility to infectious disease (75). For example, there has been recent focus on vaccine-induced non-specific innate immunity that exhibits adaptive-like characteristics, called “trained immunity” (84). BCG vaccination-induced trained immunity is shown to be associated with drastic reductions (17–37%) in LRTIs (85); however, the effects on innate immune cells appear to be transient. Epigenetic programing of monocytes by H3K4 trimethylation were detected very early in the first year but diminished by 12 months post-BCG exposure (86). Similar to BCG-induced transient changes in innate immunity, altered *intra uterine* or early-life exposures may transiently impact HEU innate immunity early in life. We do not know if the hyperinflammatory state in HEU infants early in life negatively impacts non-specific “trained immunity” or specific vaccine-induced immunity.

## Part III: Underlying Causes of Altered Immunity

### Maternal Health Status

**Figure 1:**
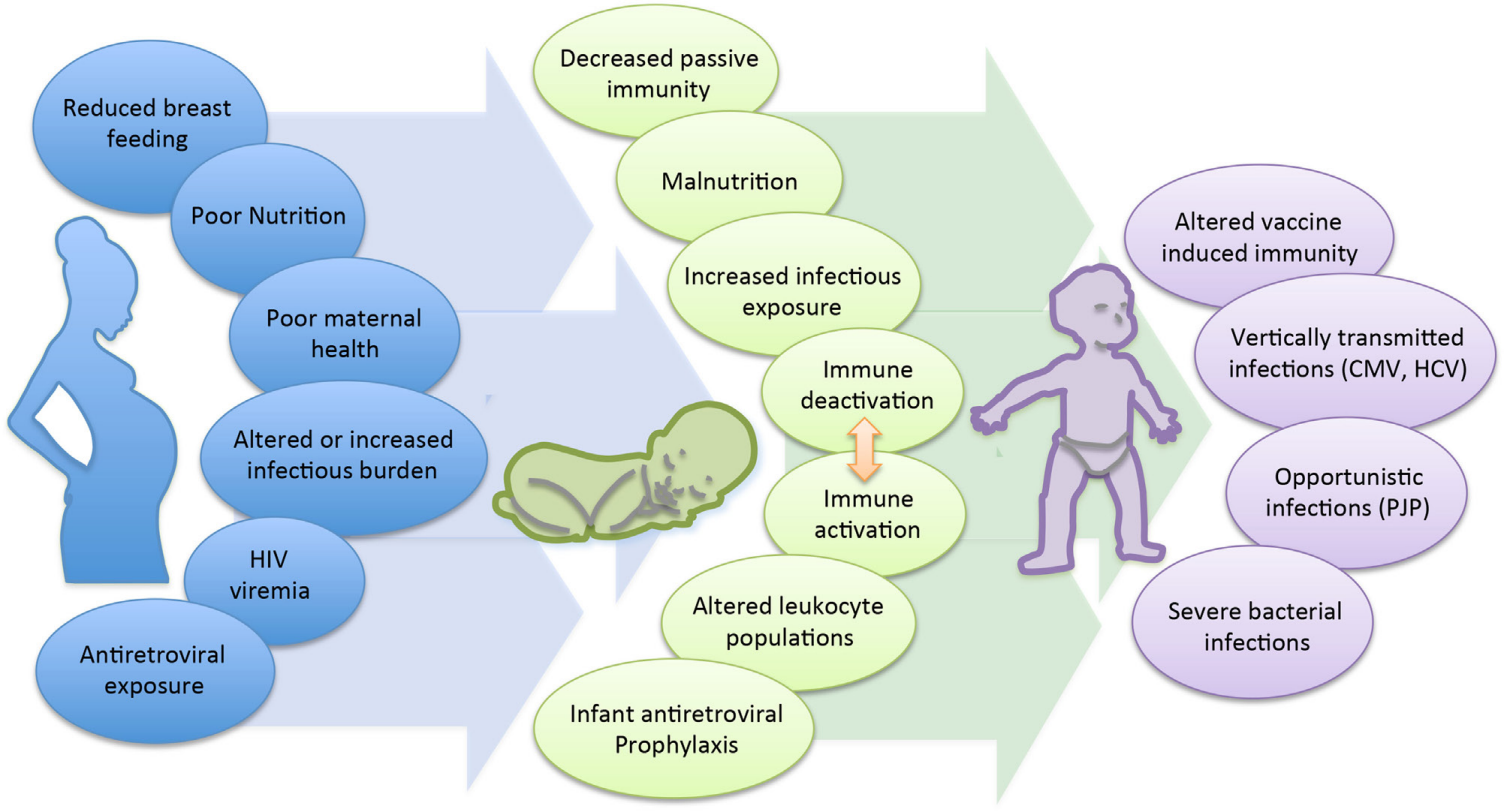
**Mechanisms contributing to poor health and survival in HEU infants**.

The cause of the immunological changes seen among the HEU is not clear, though is likely multifactorial (Figure 1). First, maternal health status and viral control is strongly suspected to play a role. Multiple studies have observed an association between maternal viral load and infant immune parameters. A prospective study of HEU infants in Mozambique reported that despite similar median levels of naive, memory, and activated CD4 and CD8 T cell counts between 1-month-old HEU and HU infants, HEU infants born to mothers with a high viral load had reduced numbers of naive CD8 and increased numbers of memory CD8 T cells compared to infants born to mothers with a lower viral load (87). Differences related to maternal viral load have also been observed at the functional level. CBMCs of HEU infants, whose mothers had a controlled viral load, produced higher levels of the anti-inflammatory cytokine IL-10 and were functionally more similar to the HU control group, while infants born to mothers with detectable viral load had reduced IL-10 and significantly higher levels of TNF-α (63). Overall, lower maternal immune status has been shown to be associated with adverse clinical outcomes in their infants. A birth cohort in Zambia found that infants of mothers with CD4 counts <350 cells/mm^3^ had a higher risk of hospitalization and mortality compared to infants of mothers with CD4 >350 cells/mm^3^ (59). A European cohort found that lower maternal CD4 count was associated with increased risk of serious bacterial, but not viral infections (35); however, increased risk of congenital and early postnatal CMV infection was seen in infants of mothers with CD4 counts <200 vs. >200 cells/mm^3^ in a US cohort (39). In the Zvitambo trial reporting on health outcomes among HEU, the incidence of oral candidiasis was significantly increased among HEU infants born to mothers with CD4 counts <200 vs. >800 cells/mm^3^ (OR: 3.91 vs. 1.91, *p* < 0.05) (34). Finally, in the two cases of HEU infants with PCP reported in Texas, the maternal CD4 counts at time of delivery were 27 and 47 cells/mm^3^, respectively (29).

### Combination Antiretroviral Therapy

Combination antiretroviral therapy (cART) during pregnancy, while essential and life-saving, may paradoxically be a contributing factor to immunological changes seen in the HEU infants. The recommended treatment for all HIV-infected pregnant women includes two nucleoside reverse transcriptase inhibitors (NRTIs) along with a protease inhibitor, for the duration of pregnancy (88). NRTI drugs induce to different degrees mitochondrial dysfunction, due to their affinity for mitochondrial gamma DNA polymerase. This affinity can interfere with mitochondrial replication, resulting in mitochondrial DNA (mt DNA) depletion and dysfunction. Aberrant histological morphology of mitochondria, mt DNA mutations, alterations in mitochondrial DNA levels in cord blood mononuclear cells, aneuploidy in cord blood cells, downregulation of select pattern recognition receptor genes, and altered secretion of soluble markers of inflammation (89–92) have all been described in both non-human primates and neonates exposed *in utero* to NRTIs (93). In the Woman and Infants Transmission (WITS) cohort study from North America, HEU infants who had had exposure to ARVs had lower total lymphocyte counts and absolute CD4 counts at 0–2 months of age, and again at 6–25 months of age, when compared to HEU who were not exposed to ARVS. Maternal CD4 counts were also significantly associated with lower infant CD4 counts in both groups (60). In the French Perinatal Cohort study, any ARV exposure was associated with lower CD4 and CD8 T cell counts in these infants, and again maternal CD4 counts <500 cells/mm^3^ were associated with lower infant CD4 counts (94). World Health Organization (WHO) guidelines recommend exclusive breast-feeding and infant ARV prophylaxis for the duration of breast-feeding (95), though little is known to date about effects of long-term ARV exposure (i.e., during the entire duration of breast-feeding) on the infant’s developing immune system.

### Feeding Patterns

The role of breast-feeding in HEU infant susceptibility to infectious diseases is not clear. On the one hand, studies have consistently shown a benefit to exclusive breast-feeding vs. formula or mixed feeding in resource-limited settings in preventing infant morbidity and mortality (96–98). This is likely due to increased transfer of maternal immune factors, including antibodies, and reduced risk of infectious exposure from potentially contaminated water sources associated with formula feeding (9, 21, 99). On the other hand, in the context of maternal HIV infection, breast-feeding presents an increased risk of HIV transmission from mother to infant (100–102). Finding the balance between reducing risk of HIV transmission, while preserving the benefits of breast-feeding, is a programmatic challenge (103). In response to the accumulated data on morbidity related to infant feeding practices, particularly in the context of ARV therapy, WHO guidelines on HIV and infant feeding practices were updated in 2010. They remain consistent with the guidelines of 2006, which recommended exclusive breast-feeding for HIV-infected mothers unless replacement feeding is acceptable, feasible, affordable, sustainable, and safe, in which case avoidance of all breast-feeding by HIV-infected mothers is recommended (104). This has resulted in different practices in resource-rich and resource-limited settings, complicating our understanding of how this may impact the developing infant’s immune system.

In resource-rich settings where exclusive formula feeding is recommended, infants may be at risk for infectious diseases in infancy due to reduced protective maternal antibodies (105, 106). However, this may be counterbalanced by expanded immunization coverage in these settings and, more importantly, increased access to health care and health services, which can mitigate this risk. In resource-limited settings, however, the recommendations for exclusive breast-feeding likely protect infants from infectious diseases-associated morbidity and mortality. Breast milk contains compounds that modulate PRR-mediated immune responses, including immunoglobulins, cytokines, antimicrobial proteins/peptides, nucleotides, and oligosaccharides (107–110). Breast milk immune composition is impacted by maternal nutritional status, exclusivity and duration of breast-feeding, and maternal or infant infections (110, 111), and maternal HIV-seropositivity does not appear to be an independent determinant of altered breast milk immune composition or quality (112). To counterbalance the increased risk of HIV acquisition during breast-feeding, the WHO recommends that all mothers remain on ARV therapy during the duration of breast-feeding (Option B+) and recommends daily antiretroviral prophylaxis for the infants in order to reduce the transmission of HIV. The protection provided through breast-feeding must be balanced against the risks associated with increased ARV exposure, and, most importantly, postnatal HIV acquisition.

### Environmental Factors

Finally, an important factor in the shaping of the innate immune system is the environment (and associated pathogens) to which a person is exposed. HEU infants may also be exposed to a different variety of pathogens in the household, as immunosuppressed HIV-positive individual(s) in the home carry a heavier burden of disease, and potentially more opportunistic pathogens (13). HEU infants may therefore have a different innate imprint from HU infants in the same setting due to increased microbial exposure in early life. As an example, increased lipopolysaccharide has been identified in the serum of infants who lack access to running water in the household (113), which can lead to chronic inflammation in the gastrointestinal system, and a gut that is more prone to bacterial translocation. Increased exposure to lipopolysaccharide can lead to immune tolerance of endotoxin; indeed, lacking access to running water correlates positively with expression of IL-10 in childhood (114). Determining if the transient diminished capacity to contain infection is mediated at least in part by non-specific environmental exposure is an important next step toward understanding morbidity and mortality in HEU infants.

## Part IV: Conclusion and Future Directions

### Summary

Across multiple cohort settings, HEU infants have been shown to have increased morbidity and mortality when compared to HU infants, and specifically higher rates of infectious diseases. While the studies reporting on microbiological diagnosis have been predominantly from South Africa, Belgium, and France, the overall reports of morbidity and mortality from infectious diseases have spanned different treatment eras (prior to and after widespread ARV availability) and geographical regions, suggesting that this is most likely related to infrastructure, research capacity, and expertise in these settings, rather than a specific geographical cluster of disease. The most commonly reported infections include invasive bacterial infections with encapsulated organisms, upper and lower respiratory tract infections, and diarrheal illnesses, most often within the first year of life, with additional case reports of PJP pneumonia. Of note, there have not been reports of increased prevalence of vaccine preventable diseases, including measles or disseminated TB, often common contributors to childhood mortality in resource-limited settings. Taken together, these findings suggest that there is likely an immunological basis for the increased susceptibility of HEU to infectious diseases in infancy.

The most consistently demonstrated difference in immune status between HEU and HU infants has been decreased maternal antibody transfer, which likely explains the increased susceptibility to encapsulated organisms in early life. However, there have been no direct studies correlating lower protective antibody levels and risk of, e.g., sepsis among HEU infants. At the same time, additional mechanisms that could explain this susceptibility to encapsulated organisms have not yet been studied, including complement deficiencies and splenic function, which may increase their susceptibility to these bacterial pathogens. Given that the susceptibility to these infections is predominantly seen during infancy and not later in life, this suggests that the humoral antibody response of HEU infants is preserved. The mechanisms that may underlie their susceptibility to opportunistic pathogens (PJP), chronic viral infections (CMV, HCV), and possibly acute viral infections (upper and lower respiratory tract infections, viral gastroenteritis), is less clear. There is some evidence of decreases in T lymphocyte number and function; however, these changes appear to be limited to select HEU infants, such as those exposed to high levels of maternal viral load or specific antiretroviral regimens. HEU infants have been shown to have a more experienced immune profile and innate immune responses, but it is not known how this may lead to increased susceptibility to disease.

### Future Directions

In this review, we have attempted to correlate the reported clinical with the immunological findings among HEU infants. From this review, it becomes clear that there remains a significant knowledge gap in understanding the immunological basis for the increased susceptibility to infectious diseases in HEU vs. HU infants. While a number of clinical, epidemiological, and surveillance-based studies have reported on infectious outcomes, these same studies have not been able to extensively study the immune profile of affected infants, likely due to the challenges in obtaining specimens and conducting immunological assays in resource-limited settings. At the same time, while a number of detailed immunological assays on a small cohort of HEU children (predominantly in resource-rich settings) have been done, there are few correlates to clinical outcomes, given the relatively small number of HEUs that become severely ill in these settings. Future work needs to be directed at directly correlating infectious disease outcomes to immunological findings among HEU infants. This would best be achieved through prospective cohort studies where detailed microbiological and immunological data would be collected in a sufficiently large number of HEU infants, with concurrent collection of detailed data on maternal immune status, *in utero* and postnatal drug exposure, and feeding patterns, in order to identify potential confounders and the relative contribution of each of these factors.

### Implications

With an estimated 1.3 million HEU children born annually, their increased susceptibility to infectious diseases and associated mortality in infancy suggests that where resources are limited, targeted interventions may be necessary for those at highest risk for adverse outcomes, such as those HEU infants born to mothers with advanced disease, highest immune suppression, and uncontrolled viremia. While standard of care in HEU follow-up varies across settings, specific health interventions could be envisioned. These include expanded vaccination coverage (including maternal immunization) to target the most identified causes of illness (pneumococcal vaccine, meningococcal vaccine, rotavirus vaccine), improved nutritional support and micronutrient supplementation, and potentially, antimicrobial prophylaxis during the first 6 months of life. A number of studies are currently underway to determine the efficacy of such interventions, including on the efficacy of antibiotic prophylaxis, the immunogenicity, and efficacy of additional vaccines in the HEU population.

## Author Contributions

CR, BR, and FK reviewed the data and drafted the initial manuscript, TK and AM oversaw the design and key contributions, and FK was responsible for the overall construct of the paper. All the authors who contributed to the interpretation of the findings were involved in the revising of the final manuscript and approved the final version to be published.

## Conflict of Interest Statement

The authors declare that the research was conducted in the absence of any commercial or financial relationships that could be construed as a potential conflict of interest.
